# Development of the digital mortality surveillance system: The Slovenian contribution to the global monitoring of mortality causes and patterns

**DOI:** 10.7189/jogh.14.03005

**Published:** 2024-01-12

**Authors:** Dalibor Stanimirovic

**Affiliations:** University of Ljubljana, Faculty of Public Administration, Ljubljana, Slovenia

## MORTUI VIVOS DOCENT (THE DEAD TEACH THE LIVING)

Mortality surveillance has been a crucial practice throughout history, serving various purposes and taking different forms. Since medieval times, it has been an essential tool for monitoring personal and legal existence. Even in modern public health, mortality surveillance remains a significant instrument, enabling the analysis of different aspects, patterns, and causes of death [1]. It plays a foundational role in monitoring mortality patterns, identifying leading causes of death, informing health policies, and improving prevention strategies. Mortality statistics, including causes of death, are crucial for monitoring population health, conducting epidemiological studies, and making international comparisons. Comprehensive mortality surveillance approaches have been recognised as valuable platforms for multi-stakeholder dialogue, resource allocation, and prioritising health programmes and initiatives [2]. Mortality statistics are widely used and serve as the primary data source for comparing health characteristics across countries. Unlike sample-based data, causes-of-death statistics encompass all deaths, thus avoiding biases and representation issues. These statistics have been employed to investigate differences in mortality levels, health prevention policies, and health care quality.

Access to comprehensive death records in a population is vital for mortality surveillance and maintaining credible public health statistics. To obtain accurate and reliable mortality statistics, medically trained and experienced doctors must carry out the death certification process, i.e. the cause of death must be determined and reported systematically and consistently to a central agency [3]. Adequate training in death certification is necessary for doctors to adhere to World Health Organization (WHO) guidelines and standards (The International Statistical Classification of Diseases and Related Health Problems – ICD) [4]. The ability of doctors to accurately diagnose the diseases and conditions leading to death depends on various factors and circumstances. In addition to its role in public health, a death certificate holds significant legal and administrative implications for the deceased’s family. It discloses the underlying cause of death, provides a legal basis for burial or cremation services, and is essential for resolving property issues and civil status matters such as insurance, pensions, and other benefits. Due to the complex and profound implications, it is crucial to complete death certificates thoroughly, accurately, and promptly [5].

The mortality surveillance system is intricate, involving multiple organisations, professional groups, and departments in the certification and registration process. These entities include doctors, coroners, the governmental bodies responsible for birth and death registration, police departments, local authorities, and national statistics agencies. Appropriately organisational, institutional, legal, administrative, and informational infrastructure must be in place to ensure a consistent sequence of activities throughout mortality surveillance. Mortality surveillance practice in Slovenia faces significant challenges in the abovementioned areas. The absence of an efficient and user-friendly digital mortality surveillance system (DMSS) further hampers existing processes and the utilisation of stakeholders' capabilities in the field of mortality surveillance. Accordingly, this text examines the international experience and domestic concerns in the field of mortality surveillance, outlines the development of the DMSS in Slovenia, and provides general guidelines for its potential implementation in other countries.

## INTERNATIONAL EXPERIENCE AND DOMESTIC CONCERNS

In the European Union (EU) and other parts of the world, completing a death certificate is a mandatory requirement for doctors or qualified individuals reporting a death. However, the accuracy of recorded causes of death has often been questioned [6]. To ensure high-quality mortality surveillance that encompasses standardised reporting and coding practices across countries, the United Nations (UN) and the WHO periodically develop protocols and guidelines for civil registration and death certification [4,7]. Additionally, the WHO and other organisations have established rules and guidelines for mortality and morbidity coding. Despite international efforts, mortality surveillance remains inadequate and fragmented in many countries, leading to vague estimates and provisional assessments of mortality patterns. Globally, only around half of the WHO member states (out of 194) have adequate mortality surveillance systems with acceptable data quality. At the same time, other countries struggle with low competencies and inconsistent coding practices [8]. Many developing countries lack comprehensive civil registration and vital statistics systems, leading to underregistration and poor data quality. Resource-constrained settings typically face challenges such as the misdiagnosis of causes of death and the underreporting of events. Factors contributing to deficient mortality surveillance and issues with data quality comprise diagnostic errors, late registration, missing information, coding mistakes, the unavailability of medical records, misinterpretations in the death certification process, and difficulties in determining the causal sequence of events leading to death [9].

Even in highly developed countries, obstacles in mortality surveillance result in inaccurate mortality reporting and statistics [10]. While the EU Member States generally have more accurate and comprehensive data, there are indications of shortcomings in the completion standards of death certificates in some developed countries as well. Variations in recording and reporting procedures between countries may affect data quality on specific causes of death. However, the European region is recognised as having the most comprehensive and up-to-date records among all regions, according to the WHO. To address these deficiencies, the European Commission emphasises the importance of high-quality mortality data and the improved international comparability of cause-of-death statistics [11]. The digitalisation of health care has become a topic of great interest in public health agendas across the EU Member States, as discussed in recent EU public health policies and reports [12]. Due to various challenges related to access to medical records, data transmission speed, the confidentiality of personal data, efficient business processes, data archiving, and the standardisation of mortality surveillance practices, several national and international public health initiatives support the implementation of DMSSs.

The use of DMSSs can undoubtedly improve the efficiency, reliability, and timeliness of mortality surveillance. However, certain challenges have to be resolved. The most common challenges in establishing comprehensive DMSSs include poor quality cause-of-death data, a lack of trained manpower in information and communication technology (ICT), high costs, coordination issues, and regular audits to control and improve data quality. In addition, clear policies and procedures are necessary to address concerns about data safety, confidentiality, and long-term archiving. Digital solutions should be introduced by structural and organisational changes and supported by integrating the DMSS with existing digital solutions in the health care system. Advances in ICT, including mobile devices, have facilitated data collection and analysis, but challenges remain regarding infrastructure and access to computers and web services in remote areas. Digital solutions can contribute to more efficient and reliable mortality surveillance, and several initiatives worldwide have been undertaken to develop effective DMSSs [13,14].

Current mortality surveillance practice in Slovenia reveals inconsistencies and shortcomings in legislation and administrative procedures, education and training, organisation, and information management. The related processes are not optimally streamlined and organised, as they rely on paper-based manual data entry and lack adequate legal and material regulation. Corner licensing, status, and jurisdiction remain unresolved, while information flows among stakeholders remain unsecured and untimely and represent serious issues. It is expected that the DMSS will address these issues to improve data flow, data quality, documentation, and archiving, as well as to unify national databases and registries. The DMSS envisions a transparent and streamlined process that includes post-mortem examination, online notification of death, access to medical documentation, decision-making on autopsies, and completing a medical cause-of-death certificate. The DMSS requires regulatory amendments, organisational changes, and business process reengineering to be effectively implemented nationally.

## DEVELOPMENT OF THE DMSS IN SLOVENIA AND GENERAL GUIDELINES FOR ITS IMPLEMENTATION

**Figure 1:**
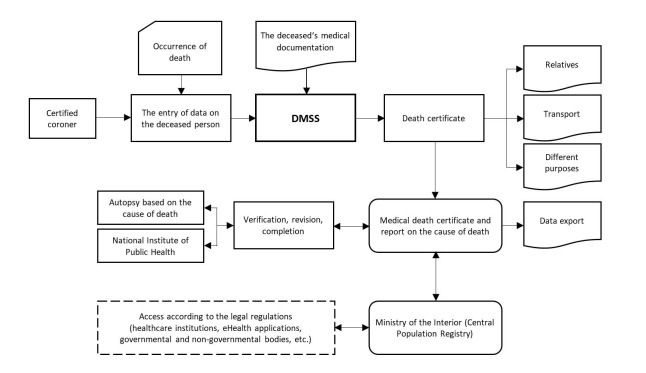
Architecture of the DMSS in Slovenia.

Death, despite being a common event in everyday life, involves complex and fast-paced actions that trigger activities across various governmental and institutional subsystems. Therefore, mortality surveillance should encompass different aspects, institutions, and activities. The developed DMSS directly addresses the identified issues and effectively resolves the current limitations related to data and information features, ensuring improved data-flow speed, the protection of personal data, access to the deceased's medical documentation, enhanced data quality and control, proper documentation and archiving, and the unification of national databases and registries. The developed DMSS is based on established standards that enable effective interoperability with eHealth solutions and other digital tools used in the Slovenian health care ecosystem (**Figure 1**).

Implementing the DMSS involves redefining the functions and relationships of key stakeholders and reconfiguring various technological, organisational, and regulatory elements to align with the declared public health objectives. Simultaneously, the DMSS advocates for re-engineered, optimised, and streamlined business processes along with significant organisational changes such as certifying coroners, implementing education and training programmes, establishing a network of coronary services, and more. These changes are dependent on amendments to regulatory and administrative provisions. The developed DMSS entails completely transforming the business model, including transitioning from paper-based forms to a web-based application. The web-based DMSS necessitates that all transactions conducted by certified coroners are digitally signed with a qualified digital certificate. This ensures a higher level of data protection throughout the unified data transmission process that bifurcates into two distinct parts: the health care part and the administrative part. This functionality aims to prevent unauthorised access to personal health data by administrative personnel and provide health care professionals with the opportunity to supplement the data on the deceased based on the conclusions and laboratory results.

The collection, storage, and processing of sensitive death surveillance data raise valid concerns regarding data privacy and related ethical considerations. Ensuring the robustness of the data privacy framework and security measures should prevent potential unauthorised access, data breaches, and cyber threats [15]. With such large health databases, other ethical questions may arise, such as data ownership, informed consent, data handling, and the potential misuse for commercial or research purposes. Disregarding the implications and value of health care data and the absence of suitable security mechanisms can undermine public trust in digitalisation initiatives, discouraging individuals from sharing their health information and using digital solutions. To maintain data security and mitigate ethical concerns around the DMSS, it is essential to incorporate principles of privacy by design, employ vigorous encryption, and adhere to strict processing rules in line with established national and international data protection regulations [16].

The existing health care regulatory frameworks worldwide often lag in adapting to the rapid changes introduced by digital solutions, leading to significant questions about the protection of personal data [17]. Consequently, sustaining effective security frameworks for safeguarding data privacy poses an ongoing challenge that necessitates continuous attention and investment. It is important to recognise and address this critical point to ensure that digitalisation projects are designed and implemented in a manner that maximises their potential benefits while minimising potential harms and inequalities. Notwithstanding this challenge, finding a delicate balance between leveraging health care data for individual and public health benefits and upholding principles of data privacy and ethical conduct remains a complex task for the future.

The DMSS has already been developed, but some of the required systemic measures and institutional actions mentioned are still in the early stages. Accordingly, the DMSS is still not in use. During the gradual implementation phase, various inconsistencies and shortcomings came to light, significantly slowing down and complicating the transition of the DMSS to its final use [18]. These challenges and systemic limitations are largely inherent to the Slovenian health care ecosystem. However, some may also appear in another country in a similar or related form [19,20]. One of the main challenges is the weak political commitment to the DMSS, as the Ministry of Health has not yet issued specific authorisations and licenses to coroners. Consequently, any medical doctor registered in Slovenia can perform the post-mortem examination service. Non-compliance with existing legislation is notable, with frequent divergence between legislation and practice, especially concerning the conditions and methods for the performance of the post-mortem examination service. A comprehensive definition for the evaluation and reimbursements of doctors’ work in connection with the post-mortem examination service is still pending. The contents of education and training programs for the post-mortem examination service have not been defined yet, highlighting a lack of clarity in this area [21,22]. Furthermore, there is a lack of provision for introducing the DMSS, as existing normative acts do not foresee the possibilities for its implementation. A certain proportion of data are of poor quality and duplicated, contributing to challenges in data management. Data from the medical records of the deceased at the time of death is also inaccessible, posing obstacles to effective information retrieval [23,24]. The inefficiency of the existing mortality surveillance practice is evident, as it does not facilitate the systematic management of pertinent documentation or its transfer to digital form.

Currently, discussions are taking place with key decision-makers and other stakeholders to garner support for preparing national recommendations in this field and facilitate the gradual implementation of the DMSS before the national roll-out of the DMSS, policy and professional consensus at the national level must be achieved regarding several open issues highlighted in the text, particularly the mentioned amendments to regulatory and administrative provisions, the professionalisation and certification of coronary services, and related expenses.

The successful development and implementation of the DMSS depends on various factors which have been identified. To pave the way for further efforts to establish efficient and high-quality DMSSs in other countries, the text provides applicable guidelines based on the lessons learned in Slovenia. These guidelines are based on a synthesis of research findings, general recommendations from the WHO [25], and Slovenia’s own experience with this demanding project:

1. Ensure policy support at the highest level by bringing together healthcare, public administration, and private sector stakeholders. Secure necessary funding and human resources and prepare comprehensive strategy documents, feasibility studies, and action plans. Promote international collaboration and provide evidence-based projections for the future development of the DMSS.
2. Mobilise all stakeholders to ensure commitment, material support, and active participation. Foster collaboration between policymakers, healthcare professionals, government officials, and ICT professionals. Establish effective communication channels within and between the project team and stakeholders.
3. Promote legislative amendments and adopt the necessary DMSS regulations. Address issues such as personal data protection, the authorisation and licensing of coroners, training and education, liability and risk management, data security, professional ethics, electronic signatures, record keeping, and data transfer.
4. Conduct a review of medical records and death certificates to assess the quality of mortality surveillance. Incorporate country-specific factors and healthcare priorities. Establish an action plan that outlines how the DMSS will contribute to addressing national healthcare priorities and facilitate the reorganisation and restructuring of the healthcare system.
5. Establish a robust evaluation framework for the DMSS, including clear objectives, benchmarking, evaluation metrics, and a combination of qualitative and quantitative indicators. Define strategic and operational measures for evaluation.
6. Select experienced managers and form a quality project team with expertise in complex ICT projects. Establish a steering committee comprising diverse experts. Assess risks and define change management strategies. Develop a well-structured project plan with clear phases, budgets, and deliverables. Assign tasks and closely monitor project progress.
7. Ensure adequate resources before each project phase and create realistic plans regarding time and finances. Define milestones and analyse capital and operating costs.
8. Maintain constant supervision and strict control over executed project tasks, ensuring that they align with substantive and temporal objectives. Monitor tasks that are currently in progress closely.
9. Enhance or establish a robust ICT infrastructure by addressing interoperability issues, improving broadband connections, adopting standard technical and medical protocols, deploying the DMSS components and technical solutions, ensuring testing and optimisation, and focusing on innovation and development.
10. Test the feasibility of the DMSS through pilot projects and gradually encourage its utilisation in healthcare institutions. Promote the adoption of the DMSS, organise education and training for coroners, issue standard practice guidelines, facilitate communication and collaboration, align business processes with medical protocols, and create a new business model that welcomes user feedback. Allow for a reasonable transition period and establish early detection and problem-solving mechanisms.
11. Provide timely updates and reports to inform stakeholders about project developments. Promote the project’s achievements to improve the acceptance of the DMSS among stakeholders. Offer comprehensive methodological explanations, user manuals, and a helpdesk. Seek support from the media, experts, and the public.

The successful execution of these guidelines depends on multiple factors and may require simultaneous or sequential activities. Coordinating these activities effectively is the most challenging task for the project management team.

## CONCLUDING COMMENTS

The increasing penetration of digital solutions in health care has raised concerns about obsolete and ineffective practices still in place. The mortality surveillance system in Slovenia has remained largely unchanged for decades, revealing various deficiencies in organisation, processes, and regulations, as well as risks related to data quality and privacy. The text suggests that implementing the DMSS could improve process integration, stakeholder coordination, and data exchange. However, the success of this project depends on the effective management of the digitalisation initiatives at the national level and their integration with health care processes.

This text does not propose a universal solution to the worldwide mortality surveillance issues. Instead, it offers valuable insights into current mortality surveillance practices and identifies factors critical to success. The findings can guide the necessary measures to transform outdated mortality surveillance practices to better meet today’s health care system and public needs. In this sense, the observations outlined in the text could also have transnational implications. They may offer applicable guidelines to versatile audiences and every country trying to establish a similar national DMSS. Despite the challenges, transforming mortality surveillance in Slovenia and elsewhere presents an opportunity to utilise available resources better, enhance policymaking, and promote public health, contingent upon effective coordination with other ecosystem factors and necessary structural reforms.
